# Balanced Adaptive Logit-Compensated Cross-Entropy and Quadratic Convolutional Network for Intelligent Fault Diagnosis Under Long-Tailed Data Distribution

**DOI:** 10.3390/e28070783

**Published:** 2026-07-10

**Authors:** Wenbin Zhang, Zikang Cao, Haijian Wu, Dewei Guo, Yasong Pu

**Affiliations:** 1 College of Mechanical and Electrical Engineering, Kunming University, Kunming 650214, China; 2 Faculty of Mechanical and Electrical Engineering, Kunming University of Science and Technology, Kunming 650500, China; caozikang@stu.kust.edu.cn (Z.C.); wuhaijian@stu.kust.edu.cn (H.W.); 3 College of Mechanical and Electrical Engineering, Honghe University, Mengzi 661199, China; guod_doug@163.com (D.G.); puyasong@uoh.edu.cn (Y.P.)

**Keywords:** long-tailed fault diagnosis, balanced adaptive logit-compensated cross-entropy loss, quadratic convolutional neural network

## Abstract

Long-tailed data are very common in industrial scenarios because equipment failures occur with a low probability, resulting in far fewer faulty samples than normal ones. However, when facing long-tailed data distributions, existing deep learning methods suffer from a significant degradation in performance and exhibit high bias. To overcome this limitation, this paper proposes a network that combines balanced adaptive logit-compensated cross-entropy loss with quadratic convolution (BALQNet) to improve diagnostic performance under long-tailed data conditions. The proposed method mainly consists of a balanced adaptive logit-compensated cross-entropy loss (BAL) and a quadratic convolution backbone. By jointly incorporating logit compensation, label smoothing, and class reweighting, BAL enhances the optimization of minority-class samples, thereby improving the classifier’s ability to distinguish different categories without introducing additional architectural complexity. Meanwhile, quadratic convolution further improves the effectiveness of feature representation learning. Finally, experiments are conducted on self-built bearing, gear, and motor datasets. The results show that BALQNet maintains strong diagnostic performance when handling long-tailed data. In addition, the ablation results provide further evidence for the effectiveness of the proposed approach.

## 1. Introduction

Rotating machinery is widely used in critical sectors of industrial production. Among these systems, core components such as bearings, gears, and motors directly affect the performance, safety, and reliability of the entire mechanical system [1,2]. Their failures are typically manifested through vibration signals, and different fault types correspond to distinct characteristic frequencies [3]. Since vibration signals contain abundant state information, numerous methods have been proposed for the fault diagnosis of rotating machinery. In general, these approaches can be categorized into two main groups: signal processing-based methods and data-driven methods [4].

Signal processing-based methods are built upon solid mathematical foundations. Time-domain signals can be transformed into frequency-domain or time–frequency representations using techniques such as Fourier transform, wavelet transform, and blind deconvolution, where spectral analysis is performed to accomplish fault diagnosis [5,6]. Although these approaches have demonstrated promising performance, they rely heavily on domain-specific knowledge and often require customized diagnostic strategies tailored to varying operating conditions, which restricts their applicability in complex industrial environments. In recent years, driven by the rapid advancement of artificial intelligence, data-driven deep learning methods have been extensively adopted for bearing fault diagnosis. As a representative model, the convolutional neural network (CNN) can automatically learn discriminative features directly from raw signals and achieve end-to-end fault diagnosis [7]. This capability significantly alleviates the limitations of traditional signal processing-based approaches and demonstrates broad application prospects.

With the continuous advancement of deep learning, various novel theoretical approaches have been proposed and applied to fault diagnosis. Zhang et al. [8] proposed a wide first-layer kernel deep convolutional neural network (WDCNN), which expands the receptive field of CNNs and demonstrates strong performance in bearing fault diagnosis tasks. Subsequently, Liao et al. [9] incorporated quadratic convolution operations into the WDCNN architecture to improve nonlinear representation ability and noise robustness, forming a quadratic convolutional neural network (QCNN) that achieved effective fault diagnosis under various noisy conditions. On this basis, Guo et al. [10] developed a physically weighted adaptive gated neural convolutional network, which further enhanced noise immunity and ensured reliable bearing fault diagnosis even under severe noise interference. Dai et al. [11] proposed a digital twin-assisted Graph Contrastive Domain Adaptation (GCDA) method, which generates cross-domain coupled twin samples and leverages graph contrastive learning to improve rolling bearing fault diagnosis under small-sample conditions. Xu et al. [12] proposed a self-supervised fault diagnosis model based on Time-Frequency Dual-Domain Prediction (TFDDP), which integrates data augmentation, a multi-attention residual encoder, and a cross-correlation smoothing matrix loss to achieve high-accuracy fault diagnosis of train traction motor bearings with limited labeled samples. Cheng et al. [13] proposed a novel knowledge distillation framework integrating improved activation functions and adaptive feature selection strategies. It effectively enhances the feature extraction and generalization abilities of the model, realizes model lightweighting, and achieves better performance in bearing fault diagnosis. Guo et al. [14] converted vibration signals into Gramian Angular Fields and combined them with deep learning algorithms to achieve cross-device gear fault diagnosis. Shao et al. [15] proposed an improved sgic-Forest deep forest model that effectively enhances the robustness of gearbox fault diagnosis under small-sample and strong-noise conditions. Xi et al. [16] proposed a fault diagnosis method integrating parameter-optimized variational mode decomposition with neural networks, enabling the adaptive extraction of fault-related features and intelligent diagnosis of gearboxes. Luo et al. [17] developed an improved symmetrized dot pattern combined with CNN to achieve high-accuracy intelligent motor fault diagnosis. Xu et al. [18] proposed a nondestructive intelligent detection method based on Cross-Sensor Self-Contrastive Learning (CSSCL), which combines cross-sensor contrastive learning, envelope spectrum analysis, and a dual-branch Transformer-MDGU encoder to achieve high-accuracy motor fault detection under different operating conditions. Moreover, deep learning architectures, including long short-term memory networks (LSTM) [19] and Transformer models [20], have been extensively utilized in rotating machinery fault diagnosis, demonstrating impressive performance. However, most of these achievements are obtained under conditions where fault data from different categories are sufficiently abundant and balanced. In practical industrial scenarios, machinery operates in a healthy state most of the time, while fault occurrences are relatively rare. As a result, in real-world scenarios, datasets often follow a long-tailed distribution, in which healthy samples significantly outnumber fault samples, leading to a significant imbalance between head and tail classes [21]. Moreover, long-tailed fault diagnosis in real industrial scenarios is further affected by variations in fault severity, changing operating conditions, and the continuous accumulation of monitoring data. These factors further increase the complexity of data distributions and make intelligent fault diagnosis more challenging. Meanwhile, a certain discrepancy still exists between artificially constructed imbalanced datasets and naturally occurring long-tailed distributions in practical industrial environments, which places higher demands on the generalization capability of fault diagnosis models. Such long-tailed distributions introduce significant challenges for deep learning models. When this imbalance is not properly addressed, the resulting models are prone to high bias and overfitting, which in turn leads to severe misclassification errors [22]. It has been reported that under class imbalance conditions with an imbalance ratio greater than 20:1, conventional methods may experience an accuracy drop of approximately 60% [23]. To alleviate the issues induced by long-tailed distributions, conventional approaches typically adopt undersampling [24] and oversampling [25] strategies. Nevertheless, these resampling-based methods may lead to the removal of a substantial portion of majority-class samples, potentially removing valuable information contained in the discarded data. To overcome the limitations of resampling techniques, researchers have introduced strategies such as contrastive learning [26], transfer learning [27], and decoupled learning [28] for fault diagnosis under long-tailed data distributions, achieving promising results. In spite of this, these approaches often require more sophisticated architectural designs, incur higher computational costs, and rely on carefully tuned optimization procedures. To overcome these limitations, this study improves the conventional cross-entropy loss and develops a novel fault diagnosis framework for long-tailed data distributions. The main contributions of this work are summarized as follows:

(1) A balanced adaptive logit compensation cross-entropy loss (BAL) is introduced to address class imbalance under long-tailed data distributions. The proposed method mitigates this issue by jointly modeling logit bias correction, gradient rebalancing, and prediction calibration. It effectively improves minority-class recognition performance without increasing the network architecture complexity.
(2) A long-tailed fault diagnosis framework, namely BALQNet, is further proposed by integrating the BAL loss with a quadratic convolutional network. By introducing the BAL loss into the quadratic convolution-based feature extraction network, the framework enables end-to-end joint optimization, thereby enhancing feature representation capability and classification performance under long-tailed distributions.
(3) Experimental validation is conducted on self-built bearing, gear, and motor datasets. The proposed method is evaluated against representative fault diagnosis models, including WDCNN, QCNN, and QResNet. Experimental results indicate that the proposed approach consistently achieves superior classification performance and enhanced robustness across different long-tailed ratios.

## 2. Theoretical Methodology

### 2.1. Convolutional Neural Network

A convolutional neural network extracts features by performing inner product operations between convolutional kernels and the input signal, followed by nonlinear activation to improve the model’s representational capacity. For an input signal *x*(*t*), the output *f*(*x*) can be mathematically formulated as:

(1) $$\sigma (f(x))=\sigma (x^{T}w+b)$$

where σ(⋅) is the nonlinear activation function, w denotes the learnable weight vector, and *b* denotes the bias term.

CNNs progressively extract hierarchical features from input data across multiple layers. Shallow layers typically learn general and low-level representations, whereas deeper layers progressively aggregate these representations into more abstract and high-level semantic features. Although CNNs have achieved remarkable success in image classification, fault diagnosis, and related domains, their fundamental operations are based on linear transformations, which inherently restrict their capability to model complex nonlinear relationships. To improve representational power and capture more sophisticated feature interactions, researchers have proposed the quadratic convolutional neural network (QCNN) [29].

### 2.2. Quadratic Convolutional Neural Network

The quadratic convolutional network extends the conventional convolutional architecture by incorporating an additional second-order term, enabling the model to capture richer nonlinear feature interactions. For an input signal *x*(*t*), the output *f*(*x*) can be mathematically expressed as:

(2) $$\begin{array}{l} \sigma ((x^{T}w^{q}+b^{q})(x^{T}w^{w}+b^{w})+{\left(x\cdot x\right)}^{T}w^{e}+n)\\ =\sigma (x^{T}w^{w}(x^{T}w^{q}+b^{q})+b^{w}x^{T}w^{q}+b^{w}b^{q}+{\left(x\cdot x\right)}^{T}w^{e})\\ =\sigma (x^{T}(w^{w}(x^{T}w^{q}+b^{q}))+x^{T}(w^{q}b^{w})+x^{T}(x\cdot w^{w}))\\ =\sigma (x^{T}(x\cdot w^{e}+w^{w}(x^{T}w^{q}+b^{q})+w^{q}b^{w}))\\ =\sigma (x^{T}(x\cdot w^{e}+w^{w}x^{T}w^{q}+w^{w}b^{q}+w^{q}b^{w})) \end{array}$$

where σ(⋅) denotes the nonlinear activation function; $w^{q}$, $w^{w}$, and $w^{e}$ represent the corresponding weight vectors; and $b^{q}$, $b^{w}$, and n are the associated bias terms.

As can be observed from Equation (2), the attention mechanism is implicitly integrated into the structure of the quadratic neuron. Specifically, the feature-weighting effect is realized through the interaction between $qttention=x\cdot w^{e}+w^{w}x^{T}w^{q}$ and $bias=w^{w}b^{q}+w^{q}b^{w}$, enabling adaptive feature modulation. Therefore, the model can achieve attention-like feature modeling without explicitly constructing the query (Q), key (K), and value (V) vectors. More specifically, the coupled dual-weight interaction in the quadratic term characterizes the interdependencies among input variables at the feature level, thereby enabling implicit correlation modeling. This mechanism enhances the representation of complex nonlinear relationships while avoiding the additional architectural complexity introduced by conventional attention modules. Consequently, the attention property becomes an intrinsic computational characteristic of the quadratic neuron rather than an externally appended structure.

As indicated in Equation (2), quadratic convolution introduces an additional second-order weight term into the computation process. Through the coupling between higher-order terms and input features, the model strengthens the representation of feature correlations and more effectively characterizes nonlinear data structures. Unlike simply stacking multiple conventional neurons, where linear weighting followed by nonlinear activation essentially forms a piecewise linear mapping, quadratic neurons construct a piecewise polynomial mapping. Compared with linear functions, polynomial functions generally possess superior capability in approximating complex nonlinear relationships [26]. Furthermore, the squaring operation is capable of characterizing the autocorrelation properties of the signal. Such an autocorrelation structure helps reinforce stable feature components while suppressing random noise interference, which allows the model to retain excellent feature extraction performance in low signal-to-noise ratio environments. Collectively, these mechanisms enhance the network’s ability to model complex nonlinear patterns and improve its generalization performance and robustness in practical engineering applications.

**Figure 1:**
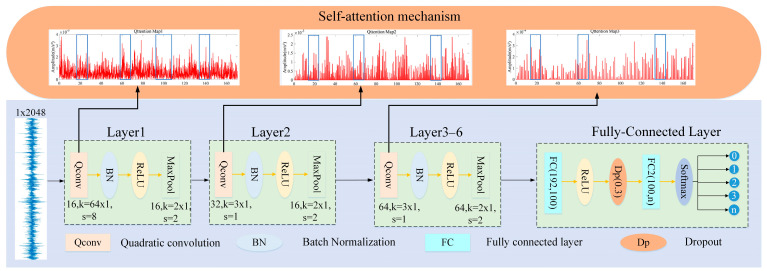
Network architecture and parameters.

Although the quadratic neuron provides a direct and flexible extension of the conventional neuron architecture, the introduction of higher-order terms inevitably brings additional nonlinear operations and learnable parameters. This increases model complexity, and to some extent, raises the difficulty of optimization during training. To solve the above problem, this work utilizes the Relinear algorithm [30] for grouped parameter optimization in the model training stage. Specifically, the parameters belonging to quadratic neurons are categorized into two sets: the linear parameter group ($w^{q}$, $b^{q}$) and the quadratic parameter group ($w^{w}$, $b^{w}$, $w^{e}$, n). In the initial phase, the linear parameter group is initialized using standard strategies. For the quadratic parameter group, the parameters are set as $b^{w}$ = 1 and $(w^{w},w^{e},n)=0$, so that the network behaves approximately as a linear model at the beginning of training, thereby improving early-stage training stability. During optimization, different learning rates, $\gamma_{q}$ and $\gamma_{w,e}$, are assigned to the two parameter groups, with the constraint that $\gamma_{q}=\alpha \gamma_{w,e}(0<\alpha <1)$. This differentiated learning rate scheme enables coordinated and progressive updates of the linear and quadratic terms throughout the training process. This grouped training strategy maintains optimization stability while effectively accelerating convergence. At the same time, it further enhances the model’s ability to capture complex nonlinear features. The backbone architecture constructed based on the above mechanism is illustrated in Figure 1.

### 2.3. Balanced Adaptive Logit-Compensated Cross-Entropy Loss

For long-tailed fault diagnosis scenarios, the sample sizes of different categories present a huge discrepancy. Since minority samples generate inadequate gradient information in the training process, standard cross-entropy loss tends to shift the decision boundary toward majority categories. This phenomenon results in severe feature distribution overlap between minority and head classes, which greatly degrades the model’s recognition capability. To tackle the aforementioned limitation, this paper develops a novel Balanced Adaptive Logit-Compensated Cross-Entropy (BAL) loss on the basis of standard cross-entropy principles. The newly designed loss unifies three mutually beneficial optimization tactics, including logit compensation, label smoothing, and class reweighting. This scheme avoids additional structural complexity and can effectively alleviate the class imbalance issue, thereby boosting the recognition accuracy and feature discrimination ability of minority samples.

The conventional cross-entropy loss function is formulated as:

(3) $$L_{CE}=-\frac{1}{N}\sum_{i=1}^{N}\sum_{c=1}^{C}y_{i,c}\log\frac{e^{\left(z_{i,c}\right)}}{\sum_{k=1}^{C}e^{\left(z_{i,k}\right)}}$$

where *N* denotes the total number of samples in a training batch, *C* represents the total number of classes, $y_{i,c}$ is the ground-truth label of the *i*-th sample for class *c*, and $z_{i,c}$ is the raw output (logit) of sample *i* corresponding to class *c*.

Standard cross-entropy loss inherently imposes an equal contribution constraint on all categories throughout model training. Nevertheless, this prerequisite is invalid for datasets with long-tailed distributions. Due to the numerical superiority of head-class samples, the optimization process preferentially reduces the loss values of majority categories. Consequently, the loss associated with minority classes is insufficiently optimized, resulting in imbalanced performance across classes and a significant degradation in overall classification effectiveness.

To overcome the aforementioned drawback, this paper develops a Balanced Adaptive Logit-Compensated Cross-Entropy (BAL) loss based on the classic cross-entropy formula. This novel loss integrates logit compensation, label smoothing and class reweighting strategies collaboratively, which remarkably boosts the recognition accuracy of minority categories and strengthens feature distinguishability while maintaining the original model’s structural simplicity.

#### 2.3.1. Logit Compensation Strategy

To alleviate the class bias caused by long-tailed data distributions, we adopt a logit compensation module in our method. Concretely, the prior probability of each category is taken as the weight coefficient to calibrate corresponding logit values prior to the Softmax operation:

(4) $${\tilde{z}}_{i,c}=z_{i,c}\;+\tau \cdot log\frac{1}{p_{c}}$$

where *p_c_* denotes the prior probability of class c, and τ is the temperature coefficient that controls the magnitude of the compensation term. By introducing this adjustment, the logits of minority classes are relatively amplified, while those of majority classes are moderately suppressed. This mechanism effectively shifts the decision boundary toward a more balanced configuration, reduces head-class dominance, and encourages the model to pay greater attention to tail classes during optimization. Importantly, the logit compensation strategy operates directly on the output layer and does not modify the backbone network structure, thereby maintaining computational efficiency while improving classification fairness under long-tailed distributions.

#### 2.3.2. Label Smoothing Strategy

In long-tailed classification tasks, the conventional one-hot encoding scheme forces the model to assign a probability of 1 to the ground-truth class and 0 to all other classes. This hard supervision may lead to overconfident predictions and reduced generalization capability, especially when minority-class samples are scarce. To alleviate this issue, a label smoothing strategy is introduced. Instead of using strict one-hot labels, the ground-truth distribution is softened by distributing a small portion of probability mass to all classes. The smoothed label for class c can be defined as:

(5) $$y_{i,c}^{LS}\;=(1-\epsilon )y_{i,c}\;+\frac{\varepsilon }{C}$$

where *ε*$\in \left[0,1\right]$ is the smoothing coefficient. By preventing the model from becoming overly confident about the majority classes, label smoothing improves generalization and enhances robustness. In long-tailed scenarios, this strategy helps reduce the excessive dominance of head classes and encourages the model to learn more discriminative representations for minority classes without modifying the network architecture.

#### 2.3.3. Class Reweighting Strategy

In long-tailed fault diagnosis tasks, the imbalance in sample quantities leads to unequal gradient contributions among classes. Majority classes dominate the optimization process, while minority classes receive insufficient updates, resulting in biased decision boundaries and degraded recognition performance. To alleviate this imbalance, a class reweighting strategy is introduced by assigning different weights to different classes in the loss function:

(6) $$\omega_{c}\;=\frac{1}{\sqrt{p_{c}}}/\left(\sum_{j=1}^{C}\frac{1}{\sqrt{p_{j}}}\right)\cdot C$$

By increasing the loss contribution of minority classes and relatively suppressing that of majority classes, the class reweighting mechanism balances gradient magnitudes during training. The weight is inversely proportional to the square root of class prior probability, i.e., minority classes obtain higher weights. This strategy effectively shifts the decision boundary toward underrepresented classes and improves overall classification fairness, while keeping the network architecture unchanged.

Combining the three aforementioned optimization tactics, the final BALCE loss function in this work is formulated as follows:

(7) $$L_{BAL}=-\frac{1}{N}\sum_{i=1}^{N}\sum_{c=1}^{C}\omega_{c}y_{i,c}^{LS}\log\frac{e^{\left({\tilde{z}}_{i,c}\right)}}{\sum_{k=1}^{C}e^{\left({\tilde{z}}_{i,k}\right)}}$$

The proposed loss unifies logit compensation, label smoothing and class reweighting within a single mathematical formulation. It effectively calibrates the decision boundaries among different categories, mitigates the prediction deviation stemming from long-tailed data distribution, restrains model overfitting, and improves the generalization performance of feature representation. In addition, this method amplifies the gradient update intensity of minority samples and further promotes the classification discrimination performance. Notably, the optimization scheme only revises the loss calculation process without adding any extra trainable parameters. Consequently, it maintains the original computational efficiency of the backbone model and possesses excellent adaptability for long-tailed fault diagnosis scenarios.

In summary, the three components of BAL serve distinct yet complementary functions. Specifically, logit compensation adjusts the classifier outputs according to class prior probabilities, thereby reducing prediction bias toward majority classes. Label smoothing alleviates model overconfidence and enhances the generalization ability and robustness of minority-class samples. Class reweighting increases the contribution of tail classes in the loss function, mitigating gradient update bias caused by class imbalance. Compared with existing representative long-tailed learning methods, BAL differs significantly in both design objectives and optimization strategies. Logit Adjustment and Balanced Softmax primarily focus on correcting classification bias induced by class priors. Class-Balanced Loss mainly alleviates class imbalance through sample reweighting. Focal Loss emphasizes hard-sample learning without explicitly considering class prior distributions. LDAM-DRW improves minority-class classification performance through class-dependent margins and a deferred reweighting strategy. In contrast, BAL simultaneously considers classification bias correction, confidence calibration, and class-balanced optimization within a unified framework, enabling these mechanisms to work synergistically and thereby improving the overall performance in long-tailed fault diagnosis tasks.

## 3. Long-Tailed Fault Diagnosis Network Model

### 3.1. Long-Tailed Fault Diagnosis System Framework

**Figure 2:**
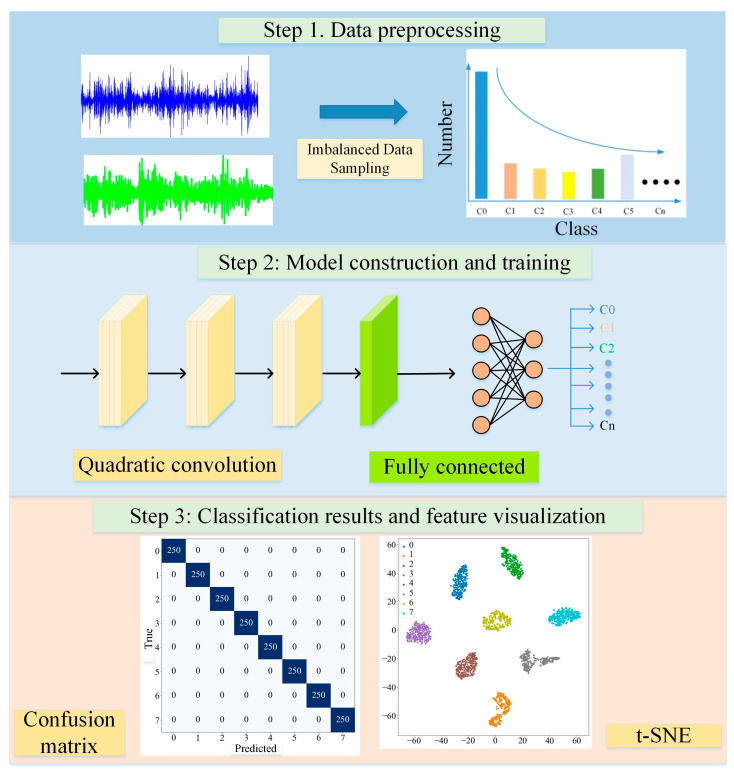
Flowchart of the proposed method.

Figure 2 depicts the overall pipeline of the presented diagnosis approach. Initially, raw vibration signals are randomly selected from the long-tailed fault dataset and input into the feature extraction module to obtain hierarchical deep feature representations. Afterward, the learned features are delivered to the classifier for fault classification, with model parameters optimized via the designed Balanced Adaptive Logit-Compensated Cross-Entropy (BALCE) loss function. In the training phase, the BALCE loss collaboratively leverages logit compensation, label smoothing, and class reweighting mechanisms. This composite optimization scheme significantly reduces the decision boundary deviation stemming from imbalanced class distribution and improves both the feature separability and classification accuracy of minority samples. Benefiting from the above designs, the established framework realizes high-efficiency and high-reliability diagnosis for long-tailed fault samples.

### 3.2. Long-Tailed Data Acquisition

In this work, multiple imbalance ratio (IBR) settings are configured to explore how data imbalance affects model prediction performance. The specific calculation formula of IBR is given as follows:

(8) $$\mathrm{IBR}=\frac{N_{n}}{N_{f}}$$

where *N_n_* is the number of normal samples and *N_f_* is the number of samples in each fault category. In this work, the IBR values are set to 5, 10, 20, and 30, respectively, to simulate varying degrees of long-tailed data imbalance.

Considering that the collected measurement signals belong to long time-series data, input samples are constructed by randomly intercepting signal segments with a length of 2048 sampling points. The overall dataset is partitioned into three subsets: training set, validation set, and test set. Specifically, the training dataset consists of 750 normal samples, and the sample size of each fault category is adjusted based on the preset imbalance ratio (IBR). In this experiment, the IBR values are set to 5, 10, 20, and 30 for comparative analysis. To achieve fair and balanced model evaluation, both the validation and test subsets adopt a fixed sample size of 250 for each category. Furthermore, unified normalization operations are applied to all samples to guarantee data consistency among different dataset partitions.

### 3.3. Evaluation Metrics

To quantitatively validate the performance of the presented approach, three commonly used evaluation indicators including accuracy, recall and F1-score are employed for experimental analysis. The detailed definitions of these metrics are illustrated below:

(9) $$\mathrm{ACC}=\frac{\sum_{i=1}^{C}\left(TP_{i}+TN_{i}\right)}{\sum_{i=1}^{C}\left(TP_{i}+FP_{i}+FN_{i}+TN_{i}\right)}$$

(10) $$F1=\frac{1}{C}\sum_{i=1}^{C}\frac{2TP_{i}}{2TP_{i}+FP_{i}+FN_{i}}$$

(11) $$\mathrm{Recall}=\frac{1}{C}\sum_{i=1}^{C}\frac{TP_{i}}{TP_{i}+FN_{i}}$$

where *C* refers to the total category quantity. *TP_i_*, *FP_i_*, *FN_i_*, and *TN_i_* correspond to the counts of true positives, false positives, false negatives and true negatives for the *i*-th category, respectively.

## 4. Experimental Analysis

To further demonstrate the superiority and feasibility of the presented algorithm, comparative experiments were carried out on three independently constructed datasets, including bearing fault, gear fault, and motor fault datasets, under imbalance ratios (IBR) of 5, 10, 20, and 30. To comprehensively evaluate the superiority of our method, we conducted comparative experiments with several state-of-the-art models that achieve excellent results in fault diagnosis tasks, namely WDCNN, QCNN and QResNet [31]. All experimental tests were executed on a computing platform with an NVIDIA RTX 4090 GPU (24 GB VRAM) and an Intel i7-13700K CPU. The proposed model was implemented using the PyTorch 2.4.0 deep learning framework in PyCharm 2024.2. The stochastic gradient descent (SGD) optimizer with a momentum coefficient of 0.9 was adopted. The initial learning rate was set to 0.01, the batch size was 32, and the number of training epochs was 200. The WDCNN model followed the above training configuration. Meanwhile, QCNN, QResNet, and the proposed method maintained identical hyperparameter configurations and further applied the parameter grouping optimization strategy presented in Section 2.2, which improves model training stability and convergence efficiency.

### 4.1. Bearing Dataset

**Figure 3:**
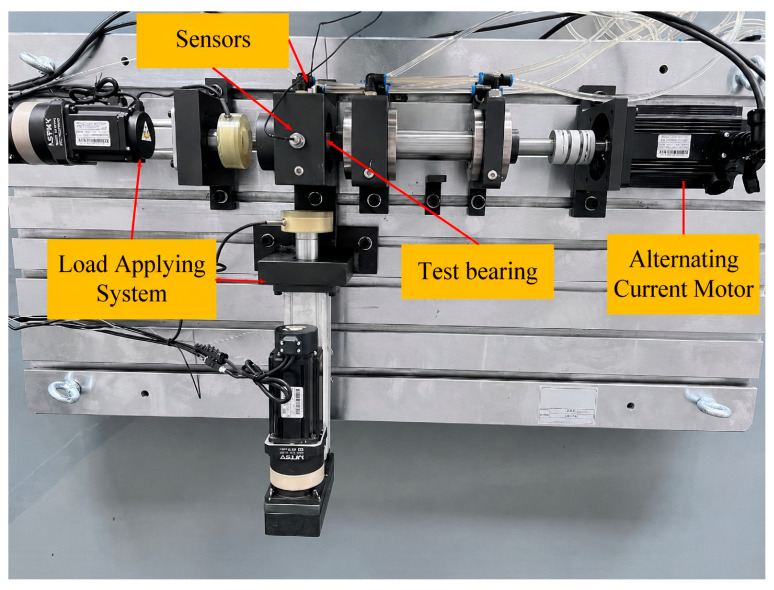
Bearing fault test rig.

The bearing dataset was collected using the experimental platform shown in Figure 3. The test rig mainly consisted of an AC motor, a bearing and its housing, and a load application device. Different fault conditions were simulated by replacing the test bearing with bearings exhibiting specific defect types. During the experiment, two acceleration sensors were mounted in the horizontal and vertical directions, respectively, to synchronously acquire vibration signals. The bearing type used in the test was SKF6206. The sampling frequency was set to 12.8 kHz, the motor speed was maintained at 1200 r/min, and the load condition was no-load. For each fault category, vibration data were continuously collected for 1 min under the corresponding operating condition. The detailed bearing health states and their corresponding classification labels are listed in Table 1.

Systematic experiments were conducted on the bearing dataset to evaluate the proposed method against the comparison models, and the results are summarized in Table 2. As shown in Table 2, when the class distribution was relatively balanced (IBR = 5:1), all four models achieved nearly perfect classification performance. This indicates that under balanced data conditions, WDCNN, QCNN, QResNet, and the proposed BALQNet are all capable of effectively accomplishing the fault identification task. However, as the degree of class imbalance progressively increases, the performance of the models begins to diverge significantly. When the IBR rises to 20:1, WDCNN exhibits the most pronounced degradation, with an accuracy drop of 0.65. Both the F1-score and recall decrease simultaneously, indicating that its representation capability for minority classes becomes severely insufficient under limited sample conditions. Similarly, QCNN demonstrates limited robustness in imbalanced scenarios. At an IBR of 30:1, its accuracy declines to 0.59, suggesting that it fails to reliably recognize long-tail categories. In contrast, QResNet shows stronger performance retention under long-tail conditions. When the IBR increases to 30:1, its accuracy remains at 0.65, significantly outperforming WDCNN and QCNN. This suggests that the combination of quadratic convolution and residual connections can partially alleviate feature degradation caused by long-tail distributions. Notably, the proposed BALQNet exhibited the strongest stability and robustness across the entire range of imbalance settings. Even under the extreme long-tail condition of IBR = 30:1, BALQNet maintained an accuracy, F1-score, and recall of 0.78, substantially surpassing the other models in overall performance. These results demonstrate that the proposed method is more effective at extracting discriminative features from minority classes and possesses superior adaptability and generalization capability in long-tail data environments.

**Figure 4:**
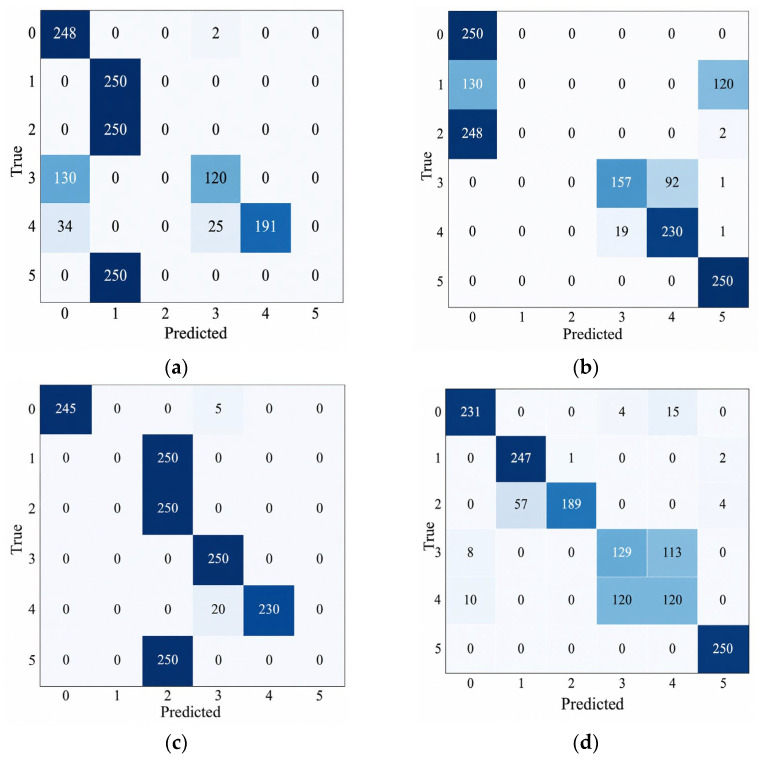
Confusion matrices of different models on the bearing dataset at IBR = 30:1. (**a**) WDCNN; (**b**) QCNN; (**c**) QResNet; (**d**) BALQNet.

To further investigate the classification behavior of each model under extreme long-tail conditions, a comparative analysis of the confusion matrices at IBR = 30:1 was conducted, as illustrated in Figure 4. It can be observed that all models maintained a high recognition rate for the healthy class (Class 0). This is primarily attributed to the sufficient number of training samples for this category, which enables the formation of stable feature representations. However, significant differences emerged among the models in their ability to classify fault categories. Specifically, WDCNN produced a considerable number of misclassifications across most fault categories (except Class 1), exhibiting a pronounced class bias toward the majority class. QCNN showed a similar tendency; although it demonstrated moderate recognition capability for certain individual categories, it still displayed an overall strong preference for majority classes. In comparison, QResNet achieved noticeable improvements in recognition performance and could accurately distinguish most fault patterns. Nevertheless, substantial confusion persisted for minority classes with extremely limited samples. Notably, BALQNet demonstrated the best performance under this severely imbalanced scenario. Except for a certain degree of confusion between Class 3 and Class 4, the remaining fault categories maintained relatively high recognition accuracy. In particular, its performance on minority classes significantly surpassed those of the other comparison models. These experimental findings further validate that BALQNet effectively mitigates class bias under long-tail data conditions and exhibits superior feature extraction capability and classification robustness.

**Figure 5:**
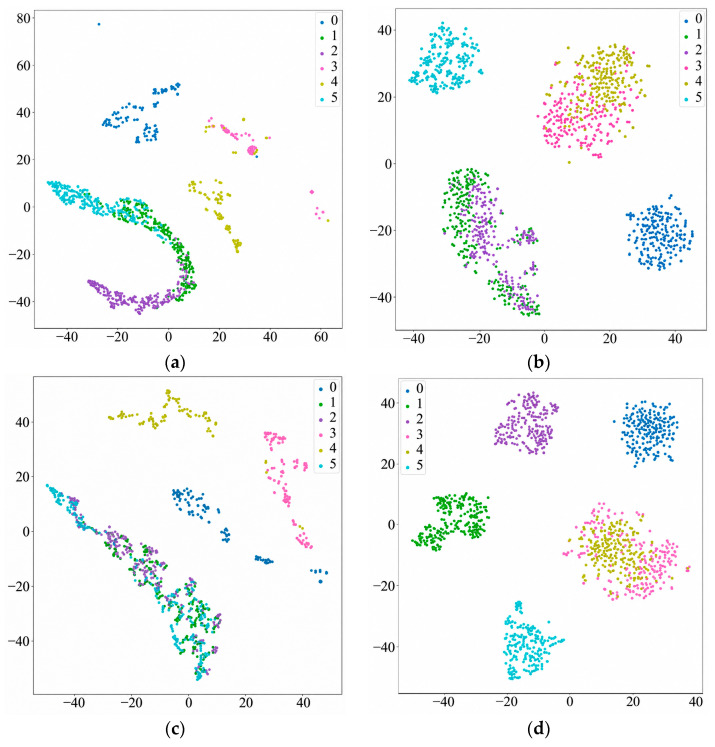
t-SNE visualizations of different models on the bearing dataset at IBR = 30:1. (**a**) WDCNN; (**b**) QCNN; (**c**) QResNet; (**d**) BALQNet.

The deep features learned by each model under the IBR = 30:1 setting were further visualized in a two-dimensional space using t-distributed Stochastic Neighbor Embedding (t-SNE), as shown in Figure 5. Overall, significant differences can be observed among the models in terms of intra-class compactness and inter-class separability under long-tail conditions. From Figure 5, it is evident that WDCNN exhibited the most disordered feature distribution. Multiple classes overlapped extensively in the low-dimensional space, and the cluster structures were highly scattered, making it difficult to form well-defined groupings. In comparison, QCNN demonstrated moderate improvement in feature organization. Certain classes began to exhibit relatively concentrated distributions; however, substantial overlap between categories still persisted, indicating limited discriminative capability. QResNet further enhanced feature separability. Most classes formed relatively distinct cluster structures, and the inter-class distances increased noticeably, resulting in improved overall clustering behavior. Nevertheless, partial inter-class mixing remained, and the cluster structures of minority classes were not yet fully stable under extreme imbalance. In contrast, BALQNet learnt significantly more compact and well-separated feature representations. The clusters were clearly distinguished, with minimal overlap between categories. This indicates that the proposed model is capable of extracting more discriminative features and maintains strong class separation ability even under severely imbalanced conditions.

### 4.2. Gear Dataset

**Figure 6:**
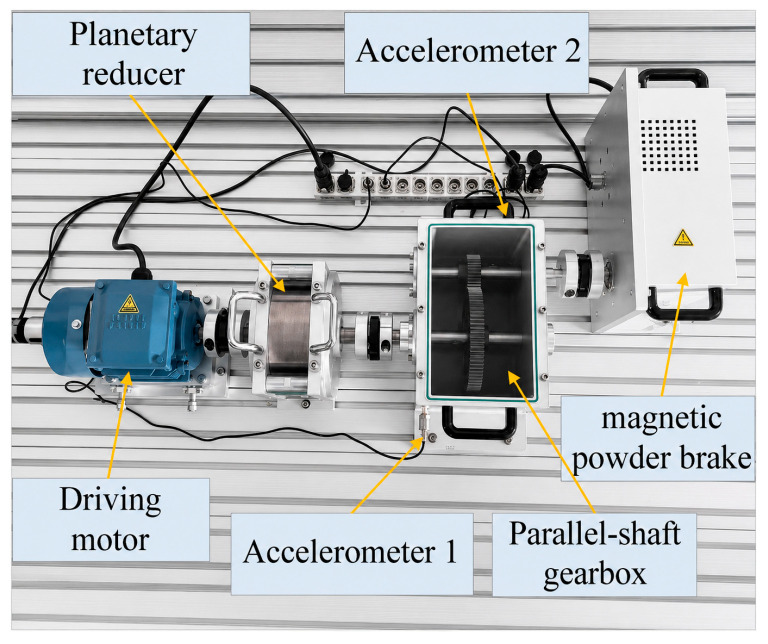
Gear fault test rig.

To further verify the generalization performance and robustness of the developed approach, additional experiments were conducted on gear vibration datasets acquired from the experimental setup illustrated in Figure 6. The overall test bench was composed of an electric motor, a parallel gearbox, a planetary gearbox and a magnetic powder brake. Different fault types were simulated by replacing the gears in the parallel gearbox. Two accelerometers were mounted on the gearbox surface to acquire vibration signals.

The sampling frequency was set to 20.8 kHz. Gear operating signals were collected under two working conditions: 20 Hz–0 V and 30 Hz–2 V. The motor was operated at constant rotational speeds of 1200 r/min and 1800 r/min, respectively, while the magnetic powder brake loads were set to 0 Nm and 2 Nm. The raw vibration signals under each gear health condition were segmented to construct the dataset. A total of 1024 samples were generated for each category, resulting in 9216 samples across the nine gear conditions. Each sample consisted of 2048 data points. The detailed gear health conditions and their corresponding classification labels are listed in Table 3.

Table 4 presents the performance variations of different models on the gear dataset as the class imbalance ratio (IBR) increases. When IBR = 5:1, all four models maintained relatively high performance, with an accuracy and F1-score both exceeding 0.91. This indicates that under mild imbalance conditions, each model retains strong feature learning and classification capability. However, as the class imbalance became more severe, the three comparison models exhibited varying degrees of performance degradation. WDCNN showed the most significant decline: its accuracy dropped from 0.92 at IBR = 5:1 to 0.75 at IBR = 10:1, further decreased to 0.67 at IBR = 20:1, and ultimately fell to 0.46 at IBR = 30:1. The F1-score correspondingly declined to 0.40, indicating pronounced class bias under high imbalance conditions. QCNN demonstrated relatively better stability than WDCNN but still exhibited a continuous downward trend as the IBR increased. At IBR = 30:1, its accuracy and F1-score decreased to 0.57 and 0.52, respectively. QResNet performed slightly better than both WDCNN and QCNN. Even at IBR = 30:1, it maintained an accuracy of 0.62 and an F1-score of 0.59. Nevertheless, its performance is still adversely affected by the scarcity of minority class samples, with noticeable degradation as the imbalance ratio grows. In contrast, BALQNet consistently demonstrated superior stability and robustness across different IBR settings. Even at IBR = 20:1, its accuracy and F1-score reached 0.81 and 0.78, respectively, significantly outperforming QCNN and WDCNN. Under the extreme long-tail scenario of IBR = 30:1, BALQNet still achieved an accuracy of 0.75 and an F1-score of 0.74, surpassing all other models. These results further validate its strong discriminative feature learning capability and enhanced minority-class recognition performance under severe class imbalance conditions.

**Figure 7:**
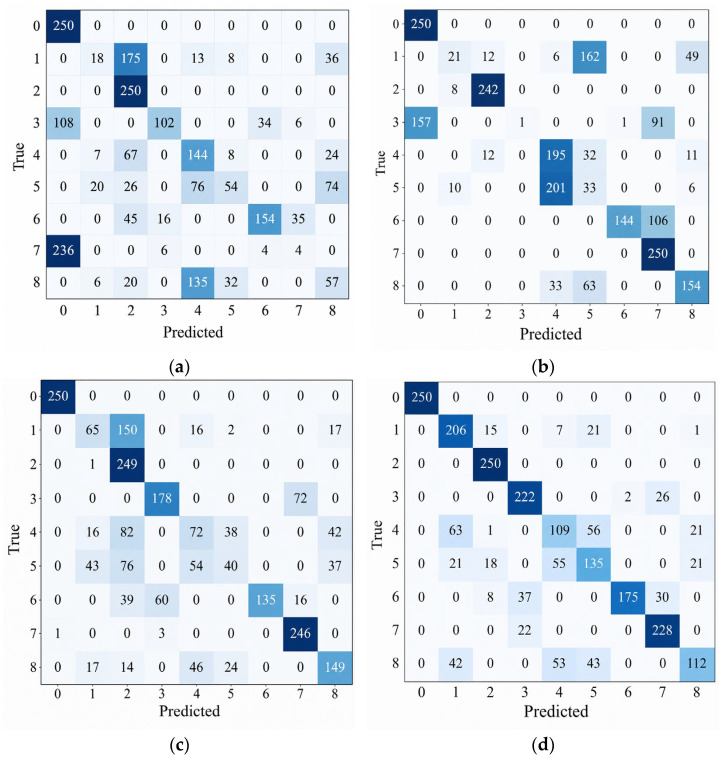
Confusion matrices of different models on the gear dataset at IBR = 30:1. (**a**) WDCNN; (**b**) QCNN; (**c**) QResNet; (**d**) BALQNet.

Likewise, to further assess the classification accuracy in extremely imbalanced scenarios, a confusion matrix analysis was conducted for the IBR = 30:1 setting, as shown in Figure 7. It can be observed that all four models achieved accurate classification for Class 0, where sufficient training samples were available. However, significant differences arise in the minority classes with limited data. WDCNN exhibited substantial misclassification across most categories except Class 0, with severe inter-class confusion, indicating that it struggles to learn reliable discriminative features under minority-class scenarios. QCNN showed moderate improvement compared to WDCNN, yet considerable confusion remained. For instance, Classes 1, 3, and 4 were frequently misclassified into majority categories, demonstrating that QCNN is still affected by majority-class bias. QResNet further enhanced feature representation capability; nevertheless, minority classes such as Classes 1, 3, and 6 still presented notable confusion, suggesting that even with deeper architectures, performance remains constrained by insufficient data. In contrast, the confusion matrix of BALQNet exhibited the clearest diagonal pattern, with consistently high classification accuracy across all categories. Notably, the recognition performance for minority classes such as Classes 1, 3, and 5 outperformed all comparative models by a notable margin. Although slight misclassifications persisted between certain classes (e.g., Classes 4 and 8), the overall confusion was substantially reduced. These results indicate that BALQNet can more effectively distinguish different fault patterns under extreme long-tail conditions.

**Figure 8:**
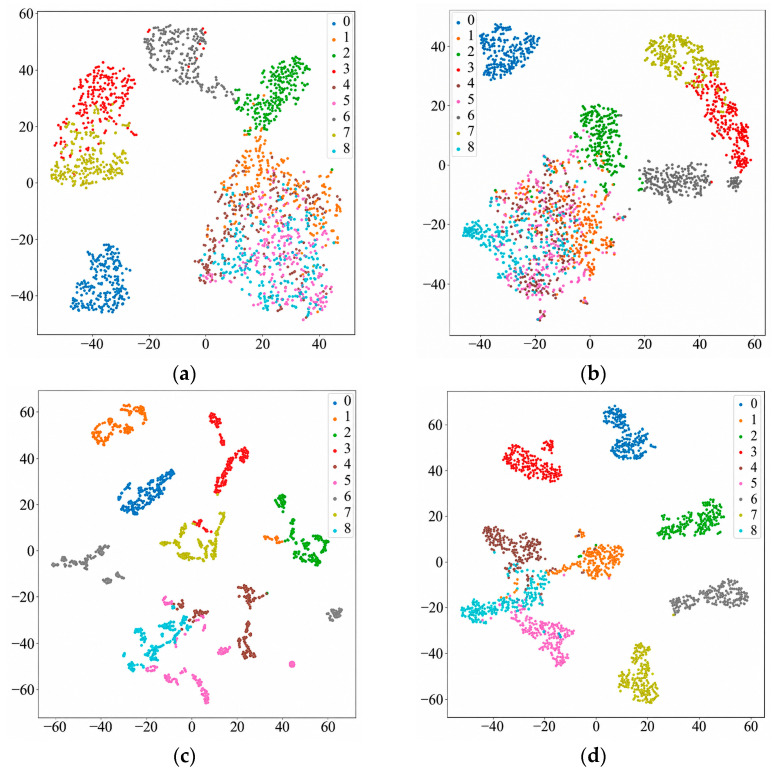
t-SNE visualizations of different models on the gear dataset at IBR = 30:1. (**a**) WDCNN; (**b**) QCNN; (**c**) QResNet; (**d**) BALQNet.

A t-SNE–based feature visualization analysis was further conducted under the IBR = 30:1 condition (as shown in Figure 8). For WDCNN, only a few categories formed relatively concentrated clusters, while the remaining classes exhibited significant overlap and intermixing, resulting in blurred inter-class boundaries. QCNN showed noticeable improvement compared to WDCNN; some categories began to demonstrate clustering tendencies, yet substantial inter-class overlap and cross-distribution still persisted overall. QResNet achieved stronger inter-class separability, with most categories forming relatively independent clusters. However, the intra-class distributions remained scattered, and the compactness within clusters was insufficient. In contrast, BALQNet produced more compact and well-structured feature distributions. Samples from each category formed clearly separated clusters with distinct boundaries and significantly reduced overlap. This demonstrates its superior feature learning capability and enhanced class discrimination ability under extreme long-tail conditions.

### 4.3. Motor Dataset

**Figure 9:**
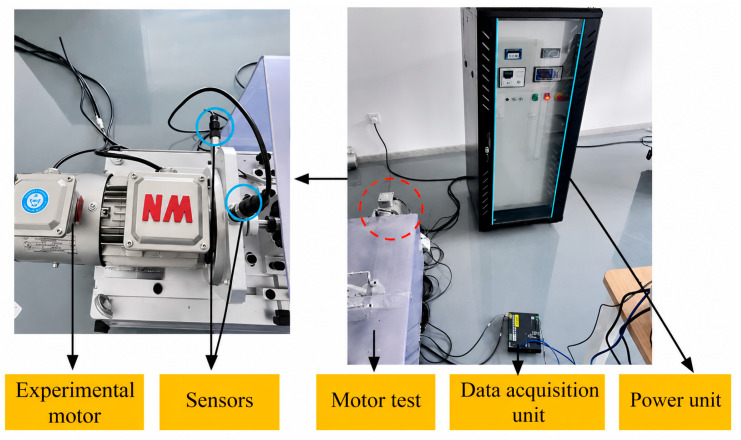
Motor fault test rig.

To further verify the generalization and effectiveness of the proposed method, simulation experiments were conducted on a motor fault implantation test rig, as illustrated in Figure 9. The experimental platform mainly consisted of a test motor, a transmission shaft, a power supply unit, and a data acquisition module. Different fault types were simulated by replacing the experimental motors, while the rotational speed and load conditions were adjusted using the control knob of the power supply unit. During the experiment, two accelerometers were installed to collect vibration signals. The sampling frequency was set to 12.8 kHz, and each data acquisition lasted for 60 s under the operating condition of 1300 r/min rotational speed and 1.72 Nm load. All collected experimental data and their corresponding labels are summarized in Table 5.

Table 6 presents the performance variations of different models on the motor dataset as the class imbalance ratio (IBR) gradually increases. The results show that under mild imbalance conditions (IBR = 5:1), all four models achieved nearly perfect classification performance, with accuracy, F1-score, and recall values close to 1.00. This demonstrates that all competing models achieved satisfactory discriminative performance under relatively balanced sample distribution conditions. However, as the imbalance ratio further increased, the three comparison models exhibited noticeable performance degradation. WDCNN showed significant sensitivity to long-tail distributions: its accuracy decreased to 0.92 at IBR = 10:1, further dropped to 0.75 at IBR = 20:1, and declined sharply to 0.50 at IBR = 30:1. QCNN demonstrated slightly smaller degradation, yet its accuracy still decreased to 0.83 and 0.64 at IBR = 20:1 and 30:1, respectively, with a corresponding decline in F1-score, indicating persistent majority-class bias. QResNet maintained relatively strong performance at IBR = 10:1 (ACC = 0.95), but its accuracy decreased to 0.85 and 0.74 at IBR = 20:1 and 30:1, respectively, revealing limitations in stability under severe imbalance. In contrast, the proposed BALQNet demonstrated the most robust performance across the entire imbalance range. At IBR = 10:1, its performance remained unaffected (ACC, F1, and recall were all equal to 1.00). Even under more extreme conditions of IBR = 20:1 and 30:1, it maintained high accuracies of 0.98 and 0.92, respectively, significantly outperforming the other three models. These results convincingly demonstrate that BALQNet possesses superior robustness and generalization capability under severe long-tail distributions, effectively mitigating majority-class bias and substantially improving minority-class recognition performance.

**Figure 10:**
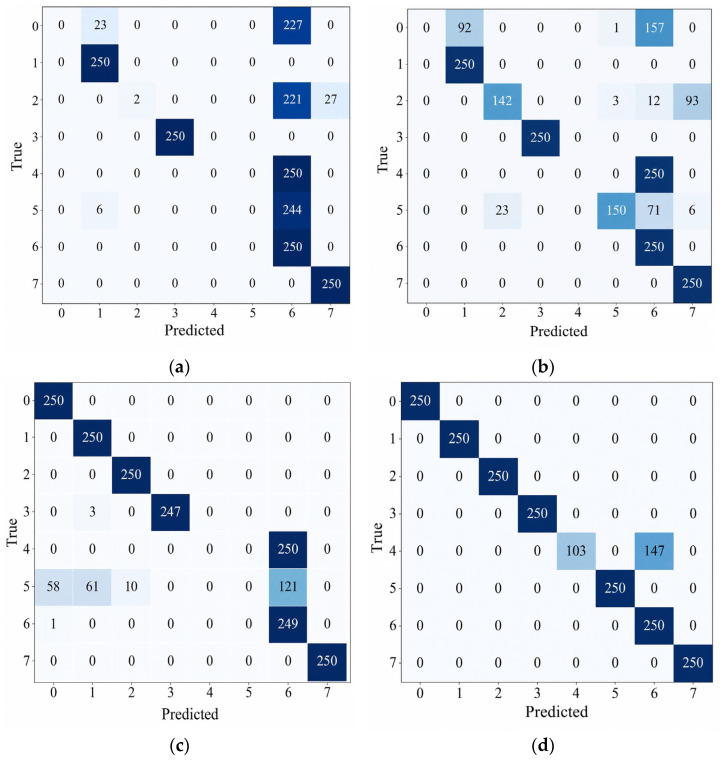
Confusion matrices of different models on the motor dataset at IBR = 30:1. (**a**) WDCNN; (**b**) QCNN; (**c**) QResNet; (**d**) BALQNet.

A confusion matrix analysis was further conducted to examine the classification performance under the extreme imbalance condition of IBR = 30:1, as shown in Figure 10. All models achieved accurate classification for Class 7, where sufficient training samples were available. However, the remaining classes were significantly affected by the long-tail distribution. Specifically, WDCNN exhibited numerous misclassifications across most categories, with severe performance degradation. QCNN similarly showed a substantial number of predictions that did not match the true labels, leading to an overall drop in classification accuracy. QResNet completely misclassified Classes 4 and 5, indicating instability under extreme imbalance. In contrast, BALQNet demonstrated much more robust performance across all classes. Misclassifications occurred only in Class 4, while all other categories were correctly classified. The outstanding performance of our model in contrast to other competitors further verifies the efficacy and generalization robustness of the proposed method in long-tailed data scenarios.

**Figure 11:**
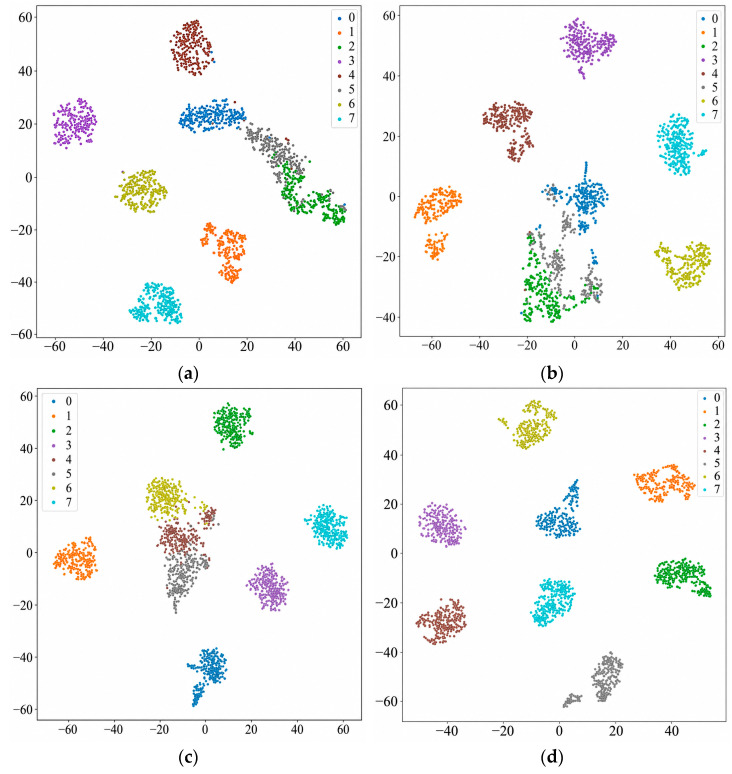
t-SNE visualizations of different models on the motor dataset at IBR = 30:1. (**a**) WDCNN; (**b**) QCNN; (**c**) QResNet; (**d**) BALQNet.

Under the extreme imbalance condition of IBR = 30:1, the feature representations of each model on the motor dataset were visualized using t-SNE, as shown in Figure 11. Compared with the bearing and gear datasets, the motor data exhibited a clearer feature structure, allowing all models to form relatively distinct class clusters. However, significant differences remained among the models. In WDCNN, overlaps between multiple classes were still evident, with some categories showing elongated, stretched patterns, indicating unstable inter-class boundaries. QCNN produced more compact clusters, but local overlaps persisted, particularly in central regions where multiple clusters were closely adjacent, reflecting residual majority-class influence under extreme imbalance. QResNet further improved feature separation, with most classes forming clear, independent clusters, although some class distances remained relatively small. In contrast, BALQNet achieved the most distinct feature clustering, with all classes forming independent, compact, and highly separated clusters with almost no overlap. This demonstrates that BALQNet effectively learns features with strong intra-class consistency and high inter-class separability under long-tail conditions, highlighting its superior discriminative capability and feature robustness.

### 4.4. Ablation Study

**Figure 12:**
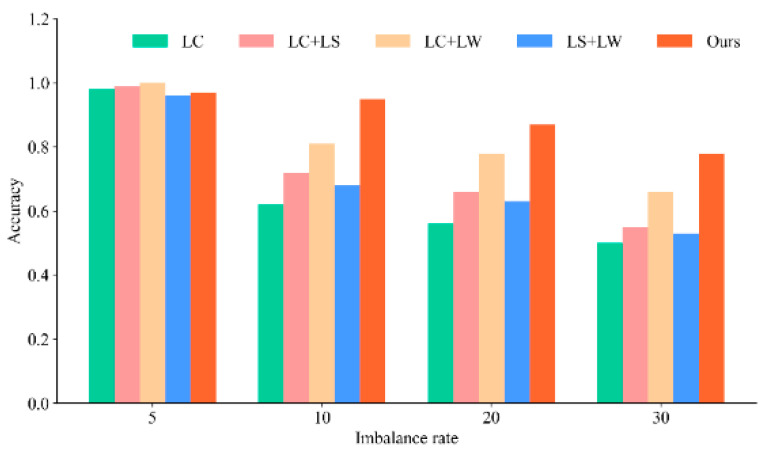
Ablation comparison experimental results on the bearing dataset.

**Figure 13:**
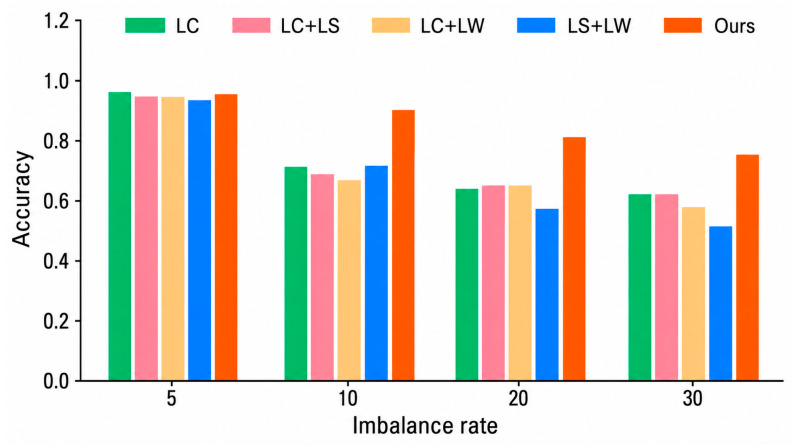
Ablation comparison experimental results on the gear dataset.

**Figure 14:**
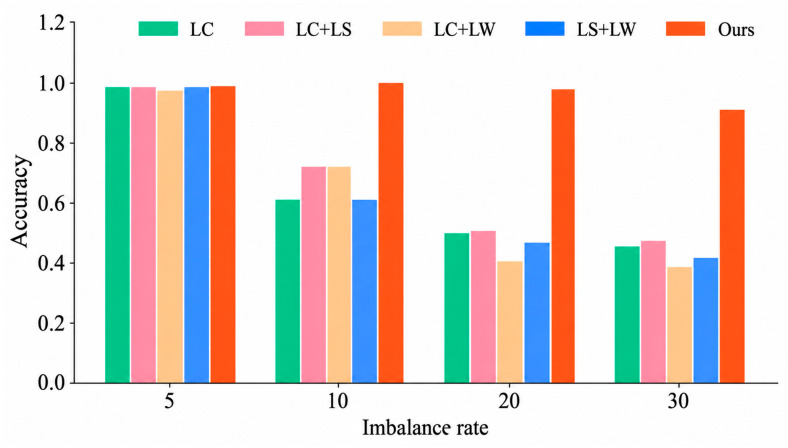
Ablation comparison experimental results on the motor dataset.

To explore the efficacy of the devised method for long-tailed fault diagnosis tasks, we carried out ablation studies with diverse loss function combinations. During the experiments, the quadratic convolutional network backbone was fixed to eliminate structural interference, and the detailed results are presented in Table 7. Meanwhile, in the previous comparative experiments on bearing, gear, and motor datasets, the effectiveness and superiority of the quadratic convolutional network were systematically validated through comparisons with baseline models such as WDCNN. Therefore, in the ablation study, the QCNN backbone was kept fixed, and the experiments mainly focused on evaluating the contributions of each component of the proposed BAL loss function. The models were evaluated on three long-tail operating condition datasets under different IBR settings, and the experimental results are presented in Figure 12, Figure 13 and Figure 14. The LC loss, widely used in long-tail scenarios in computer vision [32], achieved a reasonable performance. Incorporating LS (label smoothing) or LW (class reweighting) individually improved the diagnostic performance to some extent; nevertheless, the diagnostic performance was still restricted by inherent class imbalance, leading to marginal performance promotion. On the contrary, the collaborative integration of logit compensation, label smoothing and class reweighting yielded the optimal model performance, with the most prominent improvement observed under the extreme imbalanced scenario of IBR = 30. These experimental outcomes demonstrate that the three optimization mechanisms possess complementary functional characteristics. Combined with the quadratic convolutional backbone network, the integrated strategy can effectively alleviate the performance deterioration induced by imbalanced data distribution, thereby greatly boosting the stability and robustness of the model for long-tailed bearing fault diagnosis missions.

## 5. Conclusions

Targeting the ubiquitous long-tailed data dilemma in rotating machinery fault diagnosis, this study develops a novel BALQNet framework, which combines the Balanced Adaptive Logit-Compensated Cross-Entropy (BALCE) loss function with a quadratic convolutional backbone. The integrated BALCE loss incorporates logit compensation, label smoothing, and class reweighting strategies simultaneously. This composite optimization scheme effectively amplifies the gradient contribution of minority samples and promotes the discriminative performance of the classifier, greatly mitigating the category bias induced by imbalanced data distributions. In addition, the quadratic convolutional backbone enhances the network’s nonlinear representation ability, thereby optimizing the overall feature extraction quality for fault characteristic information. Extensive experimental validations on multiple long-tailed datasets under diverse working conditions prove that the proposed BALQNet method delivers competitive and reliable diagnostic results. Compared with existing conventional algorithms, it possesses superior robustness and cross-scenario generalization performance for long-tailed fault diagnosis tasks. Ablation studies further confirm the effectiveness of the proposed loss function. Therefore, the proposed approach provides a feasible and efficient solution for long-tail fault diagnosis in industrial applications. Nevertheless, when confronted with more extreme imbalance ratios, there remains room for further improvement in classification accuracy. Future work will focus on exploring more efficient feature enhancement and class balancing strategies to further improve diagnostic performance under severely long-tail distributions. Furthermore, practical challenges arising from fault severity variations, operating condition shifts, and continuous data accumulation remain insufficiently resolved in current research. These issues will be systematically investigated in future work to improve the robustness, adaptability, and generalization ability of fault diagnosis models in real industrial scenarios.

## Figures and Tables

**Table 1:** Bearing data types.

Number	Fault Type	Label
1	Normal	0
2	Bearing cage failure	1
3	Outer race fault	2
4	Inner–outer race compound fault	3
5	Inner race fault	4
6	Rolling element fault	5

**Table 2:** Experimental results on bearing data.

**IBR**	**5** **:1**			**10:1**		
	**ACC**	**F1**	**Recall**	**ACC**	**F1**	**Recall**
WDCNN	0.99	0.99	0.99	0.94	0.94	0.94
QCNN	1.00	1.00	1.00	0.89	0.89	0.89
QResNet	0.99	0.99	0.99	0.87	0.87	0.87
BALQNet	0.97	0.97	0.97	0.95	0.95	0.95
**IBR**	**20:1**			**30:1**		
	**ACC**	**F1**	**Recall**	**ACC**	**F1**	**Recall**
WDCNN	0.65	0.57	0.65	0.53	0.45	0.53
QCNN	0.75	0.69	0.75	0.59	0.48	0.59
QResNet	0.77	0.71	0.77	0.65	0.56	0.65
BALQNet	0.87	0.87	0.87	0.78	0.78	0.78

**Table 3:** Gear data types.

Number	Fault Type	Label
1	Normal	0
2	Broken tooth (20 Hz–0 V)	1
3	Broken tooth (30 Hz–2 V)	2
4	Missing tooth (20 Hz–0 V)	3
5	Missing tooth (30 Hz–2 V)	4
6	Root fault (20 Hz–0 V)	5
7	Root fault (30 Hz–2 V)	6
8	Surface fault (20 Hz–0 V)	7
9	Surface fault (30 Hz–2 V)	8

**Table 4:** Experimental results on gear data.

**IBR**	**5** **:1**			**10:1**		
	**ACC**	**F1**	**Recall**	**ACC**	**F1**	**Recall**
WDCNN	0.92	0.92	0.92	0.75	0.74	0.75
QCNN	0.91	0.91	0.91	0.81	0.78	0.81
QResNet	0.94	0.94	0.94	0.79	0.78	0.79
BALQNet	0.96	0.96	0.96	0.90	0.89	0.90
**IBR**	**20:1**			**30:1**		
	**ACC**	**F1**	**Recall**	**ACC**	**F1**	**Recall**
WDCNN	0.67	0.66	0.67	0.46	0.40	0.46
QCNN	0.68	0.67	0.68	0.57	0.52	0.57
QResNet	0.70	0.70	0.70	0.62	0.59	0.62
BALQNet	0.81	0.78	0.81	0.75	0.74	0.75

**Table 5:** Motor data types.

Number	Fault Type	Label
1	Current instability	0
2	Stator winding fault	1
3	Bearing outer race fault	2
4	Shaft bending	3
5	Rotor imbalance	4
6	Broken rotor bar	5
7	Rotor eccentricity	6
8	Healthy	7

**Table 6:** Experimental results on motor data.

**IBR**	**5** **:1**			**10:1**		
	**ACC**	**F1**	**Recall**	**ACC**	**F1**	**Recall**
WDCNN	1.00	1.00	1.00	0.92	0.91	0.92
QCNN	1.00	1.00	1.00	0.97	0.97	0.97
QResNet	1.00	1.00	1.00	0.95	0.95	0.95
BALQNet	1.00	1.00	1.00	1.00	1.00	1.00
**IBR**	**20:1**			**30:1**		
	**ACC**	**F1**	**Recall**	**ACC**	**F1**	**Recall**
WDCNN	0.75	0.66	0.75	0.50	0.40	0.50
QCNN	0.83	0.80	0.83	0.64	0.58	0.64
QResNet	0.85	0.85	0.85	0.74	0.67	0.74
BALQNet	0.98	0.98	0.98	0.92	0.92	0.92

**Table 7:** Ablation comparison experimental scheme table.

Method	Description
LC	Logit Compensation
LC + LS	Logit Compensation + Label Smoothing
LC + LW	Logit Compensation + Class Reweighting
LS + LW	Label Smoothing + Class Reweighting
BALQNet	Proposed method

## Data Availability

The data cannot be made publicly available upon publication because no suitable repository exists for hosting data in this field of study. The data that support the findings of this study are available upon reasonable request from the authors.
